# Hints of tRNA-Derived Small RNAs Role in RNA Silencing Mechanisms

**DOI:** 10.3390/genes3040603

**Published:** 2012-10-10

**Authors:** Maria Rosa Garcia-Silva, Florencia Cabrera-Cabrera, Maria Catalina Güida, Alfonso Cayota

**Affiliations:** 1 Laboratory of Functional Genomics, Institute Pasteur of Montevideo, Mataojo 2020, Montevideo 11400, Uruguay; E-Mails: fcabrera@pasteur.edu.uy (F.C.-C.); mcguida@pasteur.edu.uy (M.C.G.); cayota@pasteur.edu.uy (A.C.); 2 Department of Medicine, Hospital de Clínicas, Av. Italia s/n, Montevideo 11400, Uruguay

**Keywords:** tRNAs, small RNAs, RNAi

## Abstract

With the advent of new and improved high-throughput sequencing technologies in the last few years, a growing number of novel classes of small RNA, other than miRNAs or siRNA, has emerged, which appear as new actors in gene expression regulation. tRNA-derived small RNAs represent one of these novel members that are, surprisingly, among the most conserved class of small RNAs throughout evolution. They could represent the most primitive small RNA pathways from which the well-known canonical RNA silencing pathways reported in higher eukaryotes evolved. This review aims to make a compilation of the most relevant research literature in this field with the purpose of shedding light on the relation of these primitive tRNA-derived molecules with the gene silencing machinery.

## 1. Introduction

The family of non-coding regulatory RNAs (ncRNAs) has been expanding its members in view of the fact that large-scale studies of the human transcriptome revealed that a significant fraction of the genome is transcribed into these ncRNAs. Among these ncRNAs, the small ncRNAs (sRNAs) of ~20–30 nt in length have been recognized as key players in the regulation of gene expression at the transcriptional and post-transcriptional levels, which is far from being completely characterized. The best-studied members of this family are microRNAs (miRNAs), small interfering RNAs (siRNAs) and Piwi-related RNAs (piRNAs), which share members of the cell machinery for RNA interference (RNAi) gene silencing, with some exceptions. Despite the fact that they have been well described in a large number of organisms, the development of a new generation sequencing has revealed a huge amount of data that shed light on new kinds of sRNAs. These “new sRNAs” could derive from pre-existent molecules with canonical functions such as ribosomal RNAs (rRNAs), transfer RNA (tRNA), small nuclear RNAs (snRNAs), and small nucleolar RNAs (snoRNAs), which could define a group of RNAs that have a wide range of roles in gene regulation in addition to having the structural and/or functional roles for which they were originally characterized. This review aims to highlight one recently identified class of sRNAs derived from tRNA, which is frequently segregated into two main sub-classes according to their relative length and biogenesis. One group is represented by tRNA cleavage products of ~28–36 nt in length, termed generically tRNA halves (tsRNAs), mainly generated by anticodon nucleases in bacteria [1], Rny1p in yeasts [2] and angiogenin in humans [3]. An additional class of small RNAs that are ~14–22 nt in length was also reported and broadly termed tRNA fragments (tRFs) [4], which are processed by either Dicer or RNase Z and found to function similarly to miRNAs [5]. The tsRNA molecules can act as gene regulators at different levels from translational repression to post-transcriptional regulation [4,5,6,7,8]. They have been described in a wide range of organisms from prokaryotes to eukaryotes [9,10,11,12,13,14] which makes them one of the most primitive sRNA class reported. For all these reasons one complete review that contemplates all the data published up to date on tsRNAs and tRFs would be really useful in this field. Hereafter, we will focus on nomenclature matters, organisms studied and possible functions as silencing sRNAs.

## 2. Where They Live and What They Do

The tRNA-derived small RNAs have raised special interest due to the new role of tRNA molecules in cell regulatory pathways and the wide range of organisms in which they have been described. In this review, we will describe the relevance of these novel sRNAs in the regulation of gene expression and its relation to the RNA interference phenomena. A detailed list of the main characteristics of this new family is summarized in Table 1.

Despite the fact that the cleavage of tRNAs by the endoribonuclease Prrc had been observed in *E. coli* in response to phage T4 infection [15] in the 1990s, the tRFs were not taken into consideration as significant players of the gene expression regulation mechanism until recently, when additional reports suggested an RNAi-like activity for these molecules. One of these reports was the study from the ciliate *Tetrahymena thermophila*, which describes the involvement of tRFs in an early adaptive response to starvation caused by essential amino acid deprivation with a possible cell cycle regulation effect [9]. In 2008, several articles were published describing the cleavage of tRNAs in prokaryote, as well as in eukaryote cells, in order to produce tRFs as a specific cell process. Haiser *et al*. [10] associated tRFs production with the development of the bacterium *Streptomyces coelicolor*. Jöchl *et al*. [11] described the cleavage of tRNA in two halves at the anti-codon loop during conidiogenesis in *Aspergillus fumigatus*. Thomson *et al*. [13] identified a small population of tRNA and rRNA fragments that increased under certain stress conditions, being most evident by oxidative stress in *Saccharomyces cerevisiae*. The cleavage of tRNAs in response to oxidative stress occurs also in other more complex eukaryotes as *Arabidopsis* and mammalian cells.

**Table 1:** Summary of the main descriptions made to date of tRNA cleavage phenomena in different organisms and its relation with the RNAi mechanisms (N/D: Not determined).

Species	Cleavage specificity	Nuclease	Function	RNAi relation	Name
*E. coli* [1,15,16]	tRNA ^Tyr, His, Asn, Asp, Lys, Arg^	Prrc, ColicinE5 and ColicinD	Phage T4 infection	N/D	N/D
*T. thermophila* [9]	All tRNAs	N/D	Reduction of uncharged tRNA. Cell cycle progression Essential amino acids starvation	N/D	N/D
*S. coelicor* [10]	All tRNAs		Cell differentiation	N/D	N/D
*A. fumigatus* [11]	All tRNAs	N/D	Conidiogenesis Down-regulation of protein synthesis	N/D	tRNA halves
*S. cerevisiae* [2,13]	All tRNAs	Rny1	Oxidative stress	N/D	N/D
*G. Lamblia* [6]	All tRNAs	N/D	Encystation and adverse environments Down-regulation of protein synthesis	N/D	tRNA halvessitRNAs
Mammalian cells [5,8,13,17,18,19]	All tRNAs	ELAC2 DICER Angiogenin tRNaseZ Other N/D	Some stress conditions (oxidative, heat shock, UV hyperosmolarity) Down-regulation of protein synthesis	Associate to Ago2, 3 and 4. Possible miRNA/siRNA function.	tRNA halves 5' tRFs 3' CCA tRFs 3' U tRFs
*T. cruzi* [14,20]	tRNA ^Glu, Asp, Tyr, Val, Arg, His, Thr^	N/D	Nutritional stress	N/D	tRNA halves
*Drosophila* [21]	All tRNAs	PIWI	Some stress conditions	Associated to PIWI	N/D
*A. Thaliana* [13]	Al tRNAs	N/D	Nutritional and oxidative stress conditions Down-regulation of protein synthesis	ND	N/D
*C. maxima* [22]	All tRNAs	N/D	Translational Inhibition	N/D	N/D

In the same year, a particular class of tRFs was described in *Giardia lamblia*, named sitRNA for stress-induced tRNA-derived RNA (because they are produced in response to encystations which is a type of stressful condition), through cleavage in the anticodon left arm of mature tRNAs [6]. Depending on the kind of stress to which the throphozoites are exposed, *G. lamblia* produces this sitRNAs as tRNA halves. However, in contrast to tRNA halves, sitRNAs were stable during the stress response and disappeared when the throphozoites’ normal environment was reestablished. This indicates a reversible phenomenon where sitRNAs could be acting as regulatory metabolites under stress conditions and/or development to maintain down-regulated protein synthesis.

Subsequently, other studies by Thompson *et al* [13] were published concerning the existence of tRFs in plants and mammalian cells. In the case of plants, tRFs were found in *Arabidopsis thaliana* as a consequence of phosphate-starvation conditions, predominantly in roots. No enzymes have been yet related to their biogenesis and their biological function is not clear, either. However, in a work carried out in the pumpkin *Cucurbita maxima*, the authors describe that the tRFs found in this plant probably have a translation inhibition role [22].

In relation to the role of tRFs in mammalian cells, Lee *et al.* [4] studied the biological activity of a tRF (tRF-1001) that belonged to a group designated by the authors as series 1, which derived from the 3' cleavage of the pre-tRNA, in normal and cancer cell lines. In this article, the authors suggested a possible influence on cell proliferation which could take place through a mechanism distinct from miRNA/siRNA interference. Interestingly, Haussecker *et al*. [5] published an evident association between members of the RNA silencing mechanism with human tRFs of what they called type I and II tRFs. They found that both types of tRFs are associated with Argonaute proteins, preferentially Ago3-Ago4 over Ago1-Ago-2, and that experiments performed with a type I tRF (called by the author *cand45*, which is the same tRF that Lee *et al*. called tRF-1001) showed trans-silencing activity in a manner similar to miRNA/siRNA. Emara *et al.* [17] also described the cleavage of tRNA in mammalian cells by Angiogenin activity. The authors showed the production of 5' tiRNA (for tRNA-derived stress-induced RNAs) by a preferential cleavage at anticodon loop of tRNA to produce 5' and 3'-tiRNAs. Only 5' tiRNAs can inhibit translation in a translation initiation factor 2α (eIF2α) independent manner and induce stress granules assembly under certain stress conditions [8,17]. In a later work, Ivanov *et al*. [18] described that the ability of tiRNA to inhibit translation correlates with their ability to displace eIF4G/A from mRNAs. In addition, these tiRNAs were found included in an Ago2-containing complex, indicating that those tiRNAs may also interfere with miRNA-mediated regulation of gene expression. 

Along the same lines, a recent work aimed to identify the presence of alternative small RNA pathways which could contribute to the post-transcriptional control of gene expression through some RNAi o RNAi-related mechanism in the parasite *Trypanosoma cruzi*. García-Silva *et al.* [23] reported the identification and cloning of a protein characteristic of an Argonaute member distinctive from trypanosomatids (TcPIWI-tryp) and the presence of tRFs that were more pronounced in parasites undergoing nutritional stress. Surprisingly, tRFs were recruited to particular cytoplasmic granules in *T. cruzi* epimastigotes [14]. The relevance in *T. cruzi* of tRNA associations to RNAi biological activities is due to the lack of the canonical RNAi machinery in this parasite, and indeed could be using these tRFs as players of alternatives miRNA/siRNA functions.

These tRFs were also found in insects. In *Drosophila*, a recent report from Schaefer *et al*. [21], describes the generation of some tRFs, which are regulated by Dnmt2 methylation, especially under stress conditions. Moreover, *in vitro* experiments demonstrated the association of Dnmt2 with stress-related subcellular compartments and RNA processing bodies after stress conditions, indicating the involvement of this enzyme in a cell stress response mechanism, as was mentioned to occur in mammalian and parasite cells. In addition to these aforementioned tRFs, there were previous descriptions of other analogous tRNA-derived fragments in *Drosophila*, which are cleaved at the anticodon loop and are associated to Piwi proteins [24].

## 3. Classification and Biogenesis

In recent years, several groups have carried out deep sequencing assays that resulted in the description of short tRNA fragments of different sizes [4,5,11,12,25,26,27]. As mentioned above, two classes can be distinguished: those resulting from cleavage at the anticodon loop, of approximately 30–35 nt long (tsRNAs or tRNA halves), and those arising from cleavage at either the D or T loops and which are about 14–22 nt long (tRFs).

### 3.1. tRNA Halves

The tRNA halves (also called by some authors tRNA derived stress-induced fragments or tiRNAs), derived from both 3' and 5', have been described throughout the entire evolutionary scale as mentioned above, and their function as regulators of gene expression has been reported by several work groups [2,6,9,10,11,14].

**Figure 1:**
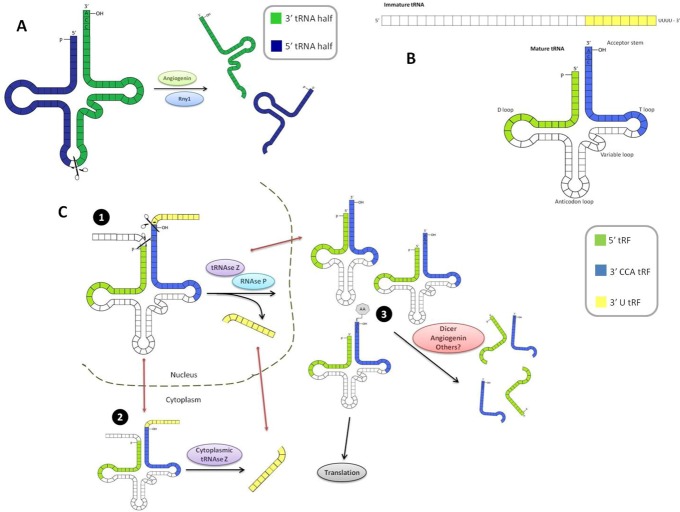
(**a**) tRNA halves generation from mature tRNAs through the action of anticodon-cleaving enzymes (Angiogenin or Rny1). 5' and 3' halves are depicted in blue and green, respectively. (**b**) Classification of small tRFs into three groups: 3' U tRFs (yellow), 3' CCA tRFs (light blue) and 5' tRFs (light green). (**c**) Scheme of the small tRFs biogenesis pathways. 3' U tRFs could be generated in the nucleus by the action of tRNAse Z during the maturation of tRNAs (1) and subsequently exported to the cytoplasm. Alternatively, immature tRNAs could be exported prematurely from the nucleus and be subjected to cleavage by a cytoplasmic pool of tRNAse Z (2). 3' CCA and 5' tRFs can be generated at any point after tRNA processing by tRNAse Z and RNAse P, from either misfolded, properly folded but not aminoacylated or aminoacylated tRNAs (3), through the action of Dicer and possibly other proteins, including Angiogenin.

tsRNAs appear as the most broadly conserved pathway of regulatory small RNAs frequently initiated after nutritional, biological or physicochemical stress in prokaryotes as well as in eukaryotes. The cleavage of tRNAs at the anticodon loop is a conserved response to some kinds of stress, such as oxidative stress and starvation, but not γ-irradiation or UV irradiation in human and yeast cells, respectively. This cleavage is carried out by Angiogenin in higher eukaryotes and by Rny1 in fission yeast, which are either secreted or sequestered proteins that can only access tRNAs during these stressful conditions [13]. Regarding their source, tsRNAs most likely come from processed tRNA transcripts, considering that the fragments so far identified lack introns, have mature 3' and 5' ends, and some include CCA sequences suggesting that, at least a variable but significant fraction are processed from mature tRNAs (Figure 1A). Nonetheless, despite mature tRNAs being the source of tRNA fragments, a significant decrease in full-length tRNAs is not registered following the biogenesis of the fragments, which implies that only a small portion of the mature tRNAs pool is involved in this process [13].

The generation of tsRNAs is not restricted to specific tRNAs, since different tRNAs are subjected to anticodon cleavage, even though to different degrees. Nevertheless, this should not be understood as a random unregulated process that targets any tRNA indistinctively [2]. Recent findings demonstrate that at least some tRNAs can be methylated by Dnmt2, protecting them from cleavage. At the same time, the generation of some tRNA halves following stress is more pronounced in cells lacking this enzyme [21]. Altogether, this points towards a regulated cellular process. Empirical evidence shows that Angiogenin-induced tRNA fragments inhibit translation initiation and suggests that they could also direct the specific degradation of mRNAs [8,18].

### 3.2. Small tRFs

Small tRFs can be easily classified into three groups, considering the region of the tRNA from which they derive. One group includes those tRFs derived from the 5' end of either mature or immature tRNA. Another group comprises those derived from the 3' end of a mature tRNA, which include the CCA sequence, and the last group comprehends those that come from the 3' end of a immature tRNA molecule or pre-tRNA, namely the 3' trailers, which characteristically have a track of uridines at their 3' end as a consequence of polymerase III transcription run-off. 

These three groups have received different names in the literature. The work from Sobala and Hutvagner [28], proposed a nomenclature based on the region of the tRNA from which the tRF comes; “5' tRFs” when derived from the 5' region, “3' CCA tRFs” for those from the 3' region of a mature tRNA (considering that they end with the CCA sequence), and finally, those from the 3' end of a pre-tRNA are called 3' U tRFs (since they end with a series of U residues). Lee *et al.* referred to these classes as the tRF-5, tRF-3 and tRF-1 series, respectively, while Haussecker *et al.* designated 3' CCA tRFs as Type I, and 3' U tRFs as Type II [4,5]. A scheme of the three classes of tRFs is depicted in Figure 1B.

### 3.3. Biogenesis of Small tRFs

The pathways and proteins involved in the generation of tRFs have been investigated by some groups. The generation of these small RNAs makes use, to some extent, of the machinery necessary for the maturation of the tRNA molecule. This is certainly the case of 3' U tRFs, which are generated by the action of tRNAse Z through the removal of the 3' trailer (one of the steps of pre-tRNA processing). This process occurs in the nucleus, yet, all tRFs are found almost exclusively in the cytoplasm, which means that after their generation they need to be exported from the nucleus [5,29]. Alternatively, a cytoplasmic pool of tRNAse Z could be responsible for the generation of 3' U tRFs from tRNAs that exited the nucleus prematurely [7].

In the case of 5' tRFs, their biogenesis is carried out by Dicer in mammalian cells, as reported by Cole *et al*. [26], through a cleavage at the D loop. However, it is known that the Dicer-independent generation of 5' tRFs takes place in *Schizosaccharomyces pombe*. The differences between the 5' tRFs generated in these two organisms, mainly in their size, support the idea that a protein other than Dicer is responsible for their production in yeast [28]. Given the absence of 5' leader sequences, 5' tRFs could be generated at any point of the tRNA processing, provided RNAse P has already removed the 5' leader. In the case of 3' CCA tRFs, their generation seems also to be Dicer-dependent, and it involves a cleavage at the T loop [5]. Considering that they include the CCA sequence at their 3' ends, their generation can only occur after the tRNA has been subjected to cleavage by both RNAse P and tRNAse Z, and the addition of CCA. It is interesting to note that, particularly in the case of 3' CCA tRFs, 5' base specificity has been shown [4]. Indeed, Lee *et al.* [4] have reported that cleavage sites are preferentially located between A/U.

Cleavage by Dicer takes place in the cytoplasm and, therefore, tRNA molecules must be imported there from the nucleus. This transport may occur at different stages of tRNA maturation: misfolded, properly folded but not aminoacylated, or fully mature [5,28]. Despite the reports of the Dicer-dependent generation of 3' and 5' tRFs, a recent study by Li *et al*. 2012 [30] in which they analyze deep-sequencing data of the wild-type and Dicer knockout cells, argues that tRFs, as a class, are independent of Dicer processing. However, they do point out that some specific tRNAs do manifest considerable differences in Dicer knockouts. These results suggested that Angiogenin, the enzyme responsible for the generation of tRNA halves, could also be involved in the generation of shorter tRFs [17]. A simplified scheme of the possible biogenesis pathways is depicted in Figure 1C. 

In spite of the somewhat contrasting findings regarding the protein machinery in charge of the biogenesis of the tRFs, the fact that they are not degradation products is a point of agreement. Thorough analysis of deep-sequencing data clearly shows that tRFs are generated following a specific cleavage pattern from either the 3' or 5' end and that sequences that comprise the anticodon loop are virtually absent [4,5,17,26]. Li *et al*. [30] suggest that similarly to the miRNA biogenesis pathway, the 3' or 5' end is preferentially maintained in the cell, leading to an asymmetrical accumulation of either one or the other. This represents one of the several characteristic shared with miRNAs or siRNAs pathways. Their size is very similar and this could be due to the fact that the enzyme implicated in their generation might be the same. It is well known that Dicer, a class III RNAse, functions as a molecular ruler, generating products of a defined length (20–25 nt) [31]. Another interesting aspect is that many tRFs have been found associated with Argonaute (Ago) proteins, fundamental for miRNA/siRNA function [5,26,32,33]. After generation by Dicer, miRNAs/siRNAs are loaded into Ago proteins and either these are responsible for mRNA degradation (if they possess slicer activity) or they recruit other proteins that act on mRNAs to silence them [34]. Taken together, this evidence argues for an implication of tRFs in gene silencing through a mechanism similar to RNAi. Empirical evidence supporting this hypothesis will be discussed in the following section.

## 4. Evidence for tRFs as Small Interfering RNAs

The sources of production of siRNAs and miRNAs can be diverse depending on the organism, but the key factors that are responsible for their effector functions are the members of the Argonaute family of proteins. Schematically, a double-stranded RNA is cleaved into fragments of approximately 22 nt by the RNase III enzyme, Dicer, of which one strand is included into an Argonaute protein complex (termed RISC, form RNA induced silencing complex) which has two chief functions: endonuclease “slicer” activity and recruitment of other proteins to target a mRNA to finally silence its expression by either translation inhibition or cleavage of the mRNA [35]. Efforts to know what kinds of sRNAs are linked to these key factors in siRNA gene silencing pathways have shed light on the diversity and complexity of this phenomenon.

Nowadays, due to the advent of new and improved high throughput sequencing techniques and bioinformatic tools, a great number of new small RNA populations different from miRNAs or siRNAs with possible relevance in gene expression regulation have been described. Among them, tRFs are one of these novel members with more evidence to support this idea. One of the most interesting studies on this topic is the finding of abundant Dicer-dependent small RNAs derived from tRNAs revealed by the analysis of deep sequencing records [26]. In this work the authors showed that tRNAs can be cleaved from a hairpin intermediate in a way similar to how miRNAs are processed and the key factor to carry out this step of the pathway also seems to be Dicer. In addition, recent studies have discovered the association of different sRNA fragments not derived from miRNA transcripts with human AGO proteins, as well [36].

Among these sRNAs associated with AGO proteins, it is possible to find, for example, fragments derived from small nucleolar RNA (snoRNA) [37] and tRNAs [5] which undergo cleavage of the precursor RNA before association with AGO proteins and formation of functional RISCs. These non-miRNA RISC complexes appear to be capable of regulating the expression of target mRNAs similar to miRNA containing RISCs. Thus, diverse sRNAs derived from cellular RNAs different from siRNAs and miRNAs could be associated with canonical pathways of gene silencing such as Dicer, Ago and RISC complexes. This association is probably competing with the classical members or regulating them at the as of yet not very well described different levels, such as acting when the cell is submitted to different stress conditions.

An interesting case was reported by Yeung *et al*. [38], which showed that tRFs associated with Ago 2 could act as siRNAs targeting a primer binding sequence in the HIV RNA genome and causing degradation of this mRNA. Additionally, Nashimoto *et al*. recently showed that, *in vitro*, the mammalian tRNA processing endoribonuclease (tRNaseZ) can cleave target mRNAs bearing a complementary binding site by using a small guide tsRNA (sgRNA) [39]. This phenomenon is called TRUE gene silencing and works on the properties of tRNaseZ guided by sgRNAs to efficiently suppress gene expression of a specific RNA.

More recently, they found that these artificial sgRNAs could be classified in four different types: 5' half-tRNA, RNA heptamer, hook RNA, and linear RNA of 14 nt long [40]. Regarding what we are concerned with, it is important to note that endogenous 5'-half-tRNA worked as sgRNA for tRNaseZ in the cells. Elbarbary and colleagues [7] showed that tRNaseZ modulates gene expression through 5'-half-tRNA. Indeed, they successfully used 5'-half-tRNA-Glu to down-regulate the level of mRNA containing its target sequence in human cells. Furthermore, their data suggested that tRNase Z guided by tRNA halves could be implicated in the p53 signaling pathway. Additionally, in a later work, they showed that these sgRNAs are able to guide tRNaseZ to cleave miRNAs and, as a consequence, down-regulating miRNA levels in the cell; this could be one of the first reports of the molecules involved in down-regulation of miRNAs. They suggest that these findings could have relevance and be employed for RNA therapy targeting diseases caused by miRNAs, such as cancer [41].

In summation, tRFs could be working as molecules that interfere with the siRNA/miRNA machinery on one hand, or, on the other hand, re-direct this machinery towards a completely different set of targets under some stress conditions or stimuli. Thus, in this respect, the machinery of siRNAs may be used by different pre-existing molecules in the cell to carry out diverse functions other than those originally described.

## 5. Conclusions

Since the discovery of the regulatory function of small non-coding RNAs in 1993 in *Caenorhabditis elegans* [42], the world of these molecules and their association to RNAi regulatory pathways is still an expanding universe. This review details what is known about a new family of tRNA-derived small RNAs originated from mature or immature tRNA molecules and discussed its relevance in the regulation of gene expression. According to recent work, these novel molecules seem to have been acting since the first steps of evolution, but despite all the findings described in this review, their biological meaning remains uncertain. These tRFs are highly diverse, displaying differences in their origin, biogenesis, size, tRNA cleavage specificity, in the abundance of their 5' or 3' halves, and in the stimuli that induce their production, as well. It is most important, however, that they seem to be involved in: (a) post-transcriptional and/or translational control functions; (b) RNAi-related processes; (c) cell cycle regulation and/or differentiation, and (d) in some cases, competing in a nonspecific manner with the RNAi machinery for determining target specificity.
